# Knowledge, Attitude, Reception, and Preventive Practices Towards Skin Photoaging Among the General Population in Jazan, Saudi Arabia

**DOI:** 10.7759/cureus.55710

**Published:** 2024-03-07

**Authors:** Ahmad Assiri, Shorooq A Hamzi, Yazeed A Hamzi, Abdulaziz Y Muyidi, Fahad M Harthi, Majed M Qaysi, Abdulmajeed A Jadah, Abdulrahman M Safhi, Alhassan H Hobani

**Affiliations:** 1 Department of Dermatology, Jazan University, Jazan, SAU; 2 Department of Pediatrics, Jazan General Hospital, Jazan, SAU; 3 College of Medicine, Jazan University, Jazan, SAU

**Keywords:** saudi arabia, jazan, sun protection, ultraviolet light, sun exposure, sunscreen, attitude, knowledge, photoaging

## Abstract

Introduction

Skin photoaging is caused by prolonged exposure to sunlight, particularly ultraviolet rays (UV). High cumulative levels of UV radiation may cause burning, photoallergic or phototoxic reactions, pigmentary changes, photoaging, and even immunosuppression and skin cancers. Therefore, this study aims to assess knowledge, attitude, reception, and preventive practices towards skin photoaging among the Jazan general population in Saudi Arabia and its determinants.

Methods

A descriptive cross-sectional study was conducted among the general population of Jazan, Saudi Arabia, who were aged 18 years and above and agreed to participate in the study. The calculated minimum sample size was 385.

An online, semi-structured, self-administered questionnaire was distributed conveniently in Google Forms through social media platforms. It included four sections: The first section was about sociodemographic characteristics. The second section assessed the smoking, exercise, and healthy diet behavior of participants and the use of sunscreen. The third section assessed the knowledge regarding the photoaging process and its preventive measures utilization using three-point Likert scale questions. The fourth section assessed attitudes towards the photoaging process and its preventive measures through three-point Likert scales.

Results

The study included 452, of which 243 (53.76%) were aged 18-30 years, 258 (57.08%) were females, and 272 (60.18%) had white skin color. Approximately 417 (92.26%) were nonsmokers. Sixty-eight percent (372) spent 1-3 hours in the sun. Social media was the primary source of information on photoaging 81 (17.92%). Around 234 (51.77%) defined photoaging correctly. Regarding sunscreen usage, 58 (12.83%) always use sunscreen, and 177 (39.16%) never use it. However, 191 (42.26%) recognized the correct sunscreen application. Approximately 233 (51.5%) and 240 (53.1%) of respondents had fair knowledge and a positive attitude regarding photoaging and sunscreen use.

Being female, pursuing university and postgraduate education, and taking information on photoaging from a physician were linked to a higher knowledge of photoaging (p<0.05). Participants who never use sunscreen had lower knowledge than those who always use it (p<0.001). None of the demographic factors was associated with the attitude towards sunscreen use (p>0.05).

Conclusion

There is a substantial gap in knowledge and preventive practices related to skin photoaging among the Jazan general population in Saudi Arabia. Gender, education level, and information sources influence knowledge levels. Targeted educational interventions are needed to enhance awareness and promote healthier practices, particularly sun exposure and photoaging prevention.

## Introduction

Skin aging is a dynamic and intricate process influenced by various internal and external factors that accumulate over time. Internal factors encompass genetic, hormonal, and natural skin aging changes. In contrast, external factors involve prolonged exposure to sun radiation, smoking, and air pollution, all recognized as prominent contributors to skin health. These factors contribute to the acceleration of skin aging through oxidative stress, which has a detrimental effect on cellular functions, including DNA replication, and the ultraviolet (UV) part of sunlight, which also causes cellular damage [1-3].

Photoaging, from prolonged exposure to sunlight, particularly UV rays, is distinct from chronological aging and manifests as premature negative changes at clinical, histological, functional, and biochemical levels [4].

Many skin diseases are known to be either initiated or aggravated by excessive UV exposure. High cumulative levels of UV radiation may cause injury to the skin cells. They may lead to tanning, burning, photoallergic or phototoxic reactions, pigmentary changes, photoaging, and even immunosuppression and skin cancers [5,6]. All these deleterious effects of UV radiation have increased the need for photoprotection. Avoiding sun exposure during peak hours of the day, seeking shade, wearing protective clothing, wide-brimmed hats, and sunglasses, and using sunscreen are the most suitable types of sun protection behaviors that have great potential to block UV radiation, delay the photoaging process, and reduce the risk of skin cancer [6-9].

Sunscreens reduce the transmission of UV radiation into the skin by reflecting, absorbing, or dispersing such emissions. Thus, sunscreen is a form of safeguard against sunlight [10]. An Indian study among participants visiting a dermatology outpatient clinic found that awareness and knowledge were poor and only 14% were using regular sunscreen [11]. Accordingly, it is essential to assess people's knowledge of the benefits and drawbacks of balanced exposure and avoidance of chronically prolonged exposure to sunlight, their attitudes towards adherence to the recommended preventive instructions for avoiding photoaging to maintain skin health, and their acceptance of the occurrence of aging in general. Therefore, this study aims to assess knowledge, attitude, reception, and preventive practices towards skin photoaging among the Jazan general population in Saudi Arabia and its determinants.

## Materials and methods

Study design, setting, and population

A descriptive cross-sectional study was conducted in Jazan, one of the 13 regions of Saudi Arabia that lie in the southwest corner directly north of the border with Yemen. We included the general population of Jazan, Saudi Arabia, who were aged 18 years and above and agreed to participate in the study.

The sample size was calculated using the Raosoft sample size calculator (Seattle, Washington, United States) [12] at a 95% confidence interval, 5% margin of error, 1.5 million population, and 50% anticipated proportion; the minimum required sample size was 385.

Data collection tools

An online, semi-structured, self-administered questionnaire was distributed conveniently in Google Forms through social media platforms. The questionnaire was adopted from previous studies [13,14]. It was reviewed by five dermatologists, and consequently, a pilot study on 20 individuals was conducted to ensure the clarity and understandability of the questionnaire. 

The questionnaire included four sections: The first section was about sociodemographic characteristics, such as age, gender, marital status, nationality, education, occupation, and skin color. The second section assessed the smoking, exercise, and healthy diet behavior of participants as well as knowledge of the term "skin photoaging," the source of information, and the use of sunscreen. The third section assessed the knowledge regarding the photoaging process and its preventive measures utilization using 13 questions coded on a three-point Likert scale (yes, no, I don't know). The fourth section contained 12 questions that assessed attitudes towards the photoaging process and its preventive measures. The questions were on a three-point Likert scale (0=disagree; 1=neutral; 2=agree).

Ethical consideration

Ethical approval was obtained from the Standing Committee for Scientific Research at Jazan University (HAPO-10-Z-001) (approval number: REC-45/04/831). Prior to the study, each patient was requested for their informed permission after being fully informed of its goals, risks, and advantages. The ability to withdraw from the study at any moment was made clear to participants. Additionally, participants are free to decline to take part in the study. Anonymity, privacy, security, and confidentiality were maintained.

Analytical plan

The data was collected, checked for completeness, and coded in an Excel sheet. The data was analyzed using IBM SPSS Statistics for Windows, Version 27.0 (Released 2020; IBM Corp., Armonk, New York, United States). Continuous variables were presented as a median and interquartile range, while categorical variables were expressed as numbers and percentages. Data normality was assessed using histograms and the Shapiro-Wilk test. For knowledge-related questions, the correct answers were coded 2, incorrect answers were coded 0, and unsure answers were coded 1, and the total knowledge score was calculated. Those scoring greater than or equal to the median were considered to have fair knowledge. The attitude score was coded as 0=disagree, 1=neutral, and 2=agree, and individuals scoring greater than or equal to the median were considered positive. The Kruskal-Wallis rank-sum and Wilcoxon rank-sum tests were employed to identify predictors of knowledge and attitude, with a significance level set at p<0.05.

## Results

The study included 452 participants, of which 243 (53.7%) fell within the 18-30 age range and 252 (57.08%) were females. The majority were singles 221 (48.01%), were Saudi 439 (97.12%), had pursued university and postgraduate studies 295 (65.27%), were students 152 (33.63%), and had white skin color 272 (60.18%) (Table 1). Regarding daily sunlight exposure, 94 (20.8%) reported being not exposed, while 309 (68.36%) spent 1-3 hours in the sun. The majority of participants were nonsmokers 417 (92.26%), didn't exercise 347 (76.77%), and didn't eat a healthy diet 262 (57.96%).

**Table 1:** General characteristics of the participants n (%)

Characteristics	N=452
Age	
18-30	243 (53.76%)
31-50	176 (38.94%)
51 and more	33 (7.30%)
Gender	
Female	258 (57.08%)
Male	194 (42.92%)
Nationality	
Non-Saudi	13 (2.88%)
Saudi	439 (97.12%)
Marital status	
Single	221 (48.89%)
Married	217 (48.01%)
Divorced	12 (2.65%)
Widow	2 (0.44%)
Education	
Less than high school	10 (2.21%)
High school	101 (22.35%)
Diploma	46 (10.18%)
University and postgraduate	295 (65.27%)
Skin color	
Beige	156 (34.51%)
Dark	24 (5.31%)
White	272 (60.18%)
Occupation	
Indoor clerks/office workers	187 (41.37%)
Outdoor work (under sun exposure)	15 (3.32%)
Students	152 (33.63%)
Not working	98 (21.68%)

Exploring awareness of skin photoaging, 325 (71.9%) of participants admitted never having heard about it. Among those who were aware, social media was the primary source for 81 (17.92%), followed by friends 17 (3.76%), lectures and books 23 (5.09%), and physicians 6 (1.33%). Participants' understanding of skin photoaging varied, with 234 (51.77%) correctly associating it with changes to unprotected skin after prolonged UV exposure, while 199 (44.03%) were uncertain about the concept. Regarding sunscreen usage, 58 (12.83%) always use sunscreen, 37 (8.19%) usually, 100 (22.12%) sometimes, 80 (17.7%) rarely, and 177 (39.16%) never. Concerning proper sunscreen application, 191 (42.26%) recognized the need to apply at least 15 minutes before sun exposure and reapply every two hours. In contrast, 161 (35.62%) thought applying immediately before exposure and reapplying every four hours was suitable (Table 2).

**Table 2:** Sun exposure-related characteristics of the participants n (%)

Characteristics	N=452
How much time are you exposed to sunlight daily?	
Not exposed	94 (20.80%)
1-3 hours	309 (68.36%)
4-6 hours	42 (9.29%)
7 hours and more	7 (1.55%)
Smoking	
No	417 (92.26%)
Yes	35 (7.74%)
Exercise	
No	347 (76.77%)
Yes	105 (23.23%)
Eating healthy diet	
No	262 (57.96%)
Yes	190 (42.04%)
Where have you heard about skin photoaging?	
I never heard	325 (71.90%)
Social media	81 (17.92%)
Friends	17 (3.76%)
Lectures and books	23 (5.09%)
A physician	6 (1.33%)
What is skin photoaging?	
It describes the changes to the unprotected skin's structure, function, and appearance after frequent or prolonged exposure to ultraviolet light	234 (51.77%)
It describes the appearance of acne on the face and body after frequent or prolonged exposure to ultraviolet light	15 (3.32%)
It describes the decrease in hair density and hair loss after frequent or prolonged exposure to ultraviolet light	4 (0.88%)
I don't know	199 (44.03%)
Do you use sunscreen?	
Always	58 (12.83%)
Usually	37 (8.19%)
Sometimes	100 (22.12%)
Rarely	80 (17.70%)
Never	177 (39.16%)
When do you have to use sunscreen?	
Applied at least 15 minutes before exposure to the sun and reapplied every two hours	191 (42.26%)
Applied immediately before exposure to the sun and reapplied every four hours	161 (35.62%)
Used before going to sleep	4 (0.88%)
I don't know	96 (21.24%)

Females had higher knowledge of photoaging than their counterparts (p<0.001). Pursuing university and postgraduate education was linked to higher knowledge than earning less than a high school education (p=0.037). Participants taking information on photoaging from a physician had higher knowledge than those taking from social media (p<0.001). Participants who never use sunscreen had lower knowledge than those who always use it (p<0.001) (Table 3).

**Table 3:** Determinants of photoaging knowledge Knowledge score: median (IQR) Kruskal-Wallis rank-sum test; Wilcoxon rank-sum test

Characteristics	N=452	p-value
Age		
18-30	21.0 (17.0, 24.0)	0.7
31-50	21.0 (17.0, 24.0)	
51 and more	21.0 (15.0, 23.0)	
Gender		
Female	22.0 (19.0, 24.8)	<0.001
Male	18.0 (13.0, 22.0)	
Marital status		
Single	21.0 (17.0, 24.0)	0.7
Married	21.0 (17.0, 24.0)	
Divorced	22.0 (20.5, 23.3)	
Widow	21.0 (18.5, 23.5)	
Education		
Less than high school	20.0 (16.3, 23.8)	0.037
High school	20.0 (16.0, 24.0)	
Diploma	19.0 (13.0, 22.0)	
University and postgraduate	21.0 (17.0, 24.0)	
Skin color		
Beige	21.0 (17.0, 24.0)	0.6
Dark	20.0 (15.8, 23.0)	
White	21.0 (17.0, 24.0)	
Occupation		
Indoor clerks/office workers	21.0 (17.0, 24.0)	0.3
Outdoor work (under sun exposure)	19.0 (13.0, 21.5)	
Students	21.0 (17.0, 24.0)	
Not working	21.0 (17.0, 24.0)	
How much time are you exposed to sunlight daily?		
1-3 hours	20.0 (17.0, 24.0)	0.5
4-6 hours	19.5 (13.5, 23.0)	
7 hours and more	20.0 (14.0, 22.0)	
Not exposed	21.0 (17.0, 23.8)	
Smoking		
No	21.0 (17.0, 24.0)	0.12
Yes	19.0 (14.0, 22.0)	
Exercise		
No	20.0 (17.0, 24.0)	0.3
Yes	21.0 (16.0, 25.0)	
Eating healthy diet		
No	20.0 (16.0, 23.0)	0.055
Yes	21.0 (17.0, 25.0)	
Source of information about skin photoaging		
A physician	25.5 (22.8, 26.0)	<0.001
Friends	21.0 (13.0, 24.0)	
Lectures and books	22.0 (17.5, 24.0)	
Social media	23.0 (20.0, 25.0)	
I never heard	20.0 (16.0, 23.0)	
Do you use sunscreen?		
Always	22.0 (19.3, 25.0)	<0.001
Usually	22.0 (20.0, 25.0)	
Sometimes	22.0 (18.0, 24.0)	
Rarely	21.0 (16.0, 23.0)	
Never	18.0 (15.0, 23.0)	

None of the demographic factors was associated with the attitude towards sunscreen use (p>0.05) (Table 4).

**Table 4:** Determinants of photoaging attitude Attitude score: median (IQR) Kruskal-Wallis rank-sum test; Wilcoxon rank-sum test

Characteristics	N=452	p-value
Age		
18-30	19.0 (15.0, 21.0)	0.9
31-50	19.0 (15.0, 21.0)	
51 and more	18.0 (15.0, 21.0)	
Gender		
Female	19.0 (15.0, 21.0)	0.8
Male	19.0 (15.0, 21.0)	
Marital status		
Divorced	21.5 (14.8, 23.0)	0.4
Married	19.0 (15.0, 21.0)	
Single	19.0 (15.0, 21.0)	
Widow	21.5 (20.8, 22.3)	
Education		
Diploma	18.5 (14.0, 21.8)	0.3
High school	19.0 (15.0, 21.0)	
Less than high school	22.0 (18.5, 23.0)	
University and postgraduate	19.0 (15.0, 21.0)	
Nationality		
Non-Saudi	19.0 (15.0, 23.0)	0.7
Saudi	19.0 (15.0, 21.0)	
Skin color		
Beige	19.0 (15.0, 22.0)	0.5
Dark	18.5 (16.8, 22.3)	
White	19.0 (15.0, 21.0)	
Occupation		
Indoor clerks/office workers	19.0 (15.0, 21.0)	0.9
Not working	19.0 (15.0, 21.0)	
Outdoor work (under sun exposure)	19.0 (13.5, 22.0)	
Students	18.5 (15.0, 21.0)	
How much time are you exposed to sunlight daily?		
1-3 hours	19.0 (15.0, 21.0)	0.9
4-6 hours	19.0 (16.0, 21.0)	
7 hours and more	15.0 (14.5, 21.5)	
Not exposed	19.5 (14.3, 22.0)	
Smoking		
No	19.0 (15.0, 21.0)	0.095
Yes	20.0 (17.0, 23.0)	
Exercise		
No	19.0 (15.0, 21.0)	0.4
Yes	18.0 (14.0, 21.0)	
Eating healthy diet		
No	19.0 (15.0, 21.0)	0.5
Yes	19.0 (15.0, 21.0)	
Where have you heard about skin photoaging?		
A physician	20.5 (16.3, 22.5)	0.7
Friends	19.0 (16.0, 21.0)	
I never heard	19.0 (15.0, 21.0)	
Lectures and books	20.0 (16.0, 22.0)	
Social media	19.0 (15.0, 21.0)	
Do you use sunscreen?		
Always	19.0 (14.3, 22.0)	0.2
Usually	20.0 (16.0, 23.0)	
Sometimes	18.0 (14.8, 20.0)	
Rarely	19.0 (15.0, 20.0)	
Never	19.0 (16.0, 21.0)	
Knowledge of photoaging		
Fair knowledge	19.0 (15.0, 21.0)	0.4
Unfair knowledge	19.0 (15.0, 21.0)	

**Figure 1:**
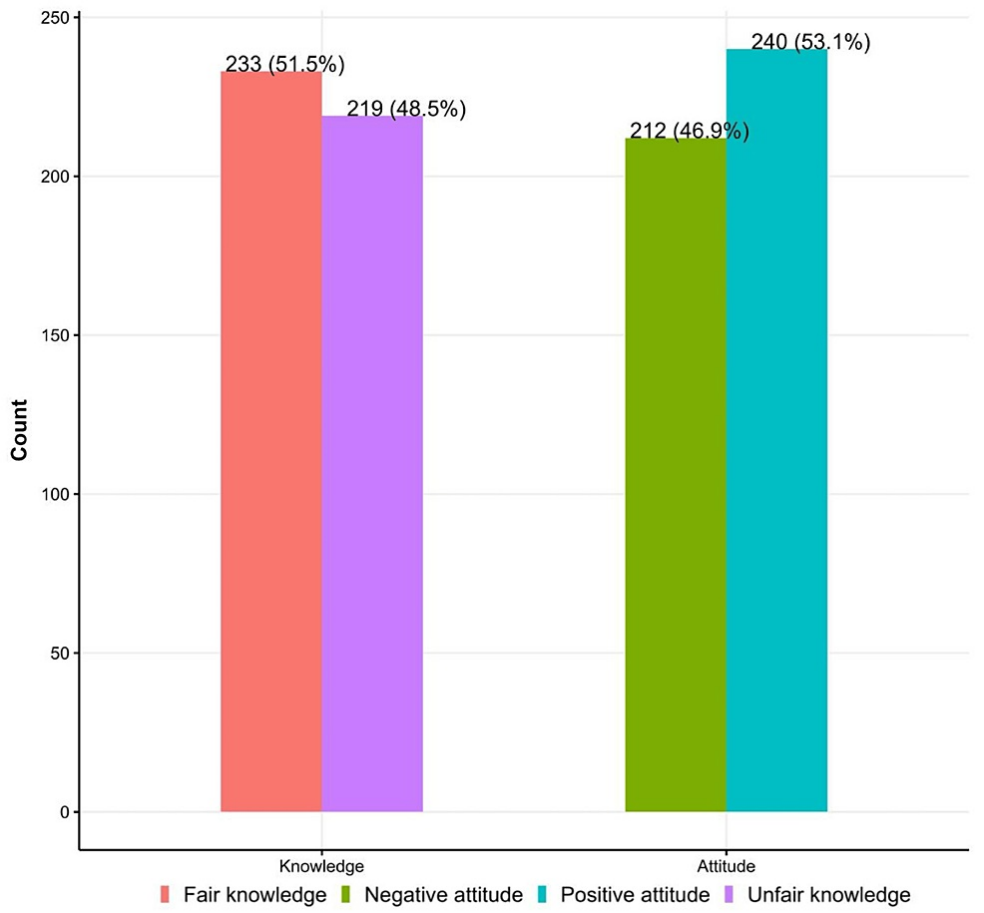
Knowledge and attitude regarding photoaging

Approximately 233 (51.5%) and 240 (53.1%) of respondents had fair knowledge and a positive attitude regarding photoaging and sunscreen use (Figure 1).

## Discussion

Knowledge, attitudes, and practices towards photoaging reveal important insights into public awareness and behaviors related to skin health. Skincare encompasses various practices. Skin issues significantly impact individuals' quality of life and health [15], particularly in older adults, due to structural changes in the aging skin [16]. Essential skin functions include water conservation, electrolyte and protein retention [17], acting as a barrier between internal and external environments [18], sweat and vitamin D production, UV absorption, temperature regulation, and autonomic/immunologic actions [19].

This study explored the knowledge, attitudes, and practices of participants from Saudi Arabia regarding photoaging. The results show that much needs to be done to provide more education for individuals within these population groups that should help to improve their attitudes towards skincare and encourage them to institute practices that will offer them opportunities for healthier lives.

Four hundred and fifty-two participants were enrolled in this study, out of which 285 (57%) were females. Regarding daily sunlight exposure, 94 (21%) reported being not exposed, while 309 (68%) spent 1-3 hours in the sun. Skin damage from excessive sunlight can come in several forms: skin cancer, photoaging, rosacea, and chronic discoid lupus erythematosus [20]. Studies have shown that adverse effects on skin health can result from exposure to 2,4-dichlorophenoxyacetic acid and paraquat [21] or organochlorine pesticides such as dichlorodiphenyltrichloroethane (DDT) [22]. Such adverse effects can include the development of contact dermatitis, urticaria, erythema multiforme, ashy dermatosis, chloracne, hypopigmentation of the skin, and skin cancer [23].

In terms of knowledge, the findings suggest a varying degree of understanding among individuals regarding the impact of sun exposure on skin aging. Approximately 233 (51.5%) demonstrated a good level of knowledge about photoaging and sunscreen use. Analysis revealed that 325 (72%) of participants had never heard about photoaging. On the other hand, among those who were aware, social media was the primary source for the majority. Participants' understanding of skin photoaging varied, with 233 (52%) demonstrating a solid understanding of the connection between UV radiation and premature aging, highlighting the importance of education and awareness campaigns.

Regarding attitude, 240 (53.1%) of respondents showed a positive attitude regarding photoaging and sunscreen use. Attitudes towards photoaging appeared to be influenced by cultural perceptions and societal norms. While some individuals expressed a proactive attitude, adopting sun-protective measures, others displayed a more complacent approach, possibly due to misconceptions or a lack of perceived severity.

Sunlight and UV radiation are primary factors in skin problems [24]. Protective measures, such as limiting outdoor activities during peak UV hours, seeking shade, wearing suitable hats and clothing and sunglasses, and using sunscreen, play a crucial role in preventing and safeguarding the skin against disorders [25,26]. Practices related to photoaging varied widely, with 58 (13%) always using sunscreen, compared to 37 (8.2%) who use it on a usual basis. This is lower than the findings from a previous study in Peru, where of the total number of users of photoprotection, 38.4% used these products daily, while 61.6% used them only occasionally [27]. This variation could be attributed to differences in socioeconomic status, level of education, and perception between the participants. Concerning proper sunscreen application, 191 (42%) recognized the need to apply at least 15 minutes before sun exposure and reapply every two hours. In contrast, 161 (36%) thought applying immediately before exposure and reapplying every four hours was suitable. This finding gives an insight into a notable discrepancy between knowledge and behavior. Despite knowing the harmful effects of sun exposure, many participants reported inconsistent sun protection practices. This suggests a potential gap between awareness and actual implementation of preventive measures.

Factors influencing knowledge, attitudes, and practices towards photoaging could include age, socioeconomic status, and access to information. Younger participants may exhibit a more carefree attitude, while those with higher socioeconomic status might prioritize skincare practices. Correlation testing revealed that females had higher knowledge of photoaging than their counterparts (p<0.001). A similar study revealed that women were more knowledgeable regarding sun protection (p=0.001) [27]. Not only knowledge but also practice was influenced by gender, where a previous study conducted in Saudi Arabia found that females used sunscreen and protection methods more frequently than their male counterparts [28]. This can also be attributed to the finding of a previous study where using skincare products was significantly associated with the female gender (p<0.001) [29].

Pursuing university and postgraduate education was linked to higher knowledge than earning less than a high school education (p=0.037). Participants taking information on photoaging from a physician had higher knowledge than those taking from social media (p<0.001). This is supported by the results of a previous study where knowledge regarding sun protection was more evident in individuals with university/college education (p<0.001) [27], indicating a positive correlation between educational level and knowledge regarding photoaging.

Addressing the gap between knowledge and practices is crucial for effective public health interventions. Educational campaigns targeting specific demographics and emphasizing the tangible benefits of sun protection could contribute to a positive shift in attitudes and behaviors towards preventing photoaging. Further research is needed to explore the long-term impact of interventions on knowledge, attitudes, and practices and ultimately reduce the prevalence of photoaging in the population.

Strengths of the study

A limited number of Saudi studies were published in this scope; hence, this study is considered a valuable base for evidence. Another strength of this study is that it included participants from variable demographic backgrounds and socioeconomic status, which would aid the authorities in dealing with the issue from all aspects. 

Limitations of the study

The study was not without limitations. The fact that it was done within a specific setting may have determined a highly selected group of cases. It may, therefore, be difficult to generalize the findings to the total community in Saudi Arabia. Larger numbers of respondents would have improved the statistical significance of the results.

## Conclusions

The study showed a good level of knowledge and favorable attitude towards photoaging and sunscreen use among half of the participants. A significant association between gender and knowledge was noticed, with females having a higher knowledge of photoaging. In addition, a high level of education was linked to a higher knowledge than those who earned less than a high school education. Serial and frequent studies on the issue should be conducted and funded to generate more evidence and data. In addition, educational campaigns, medical missions, and media recruitment should be held to increase community awareness of such issues, and attempts to enhance patients' practices should be arranged. Examples are offering skin care products for affordable prices or even setting shade umbrellas in public spaces.
